# Assessing the Level and Determinants of COVID-19 Vaccine Confidence in Kenya

**DOI:** 10.3390/vaccines9080936

**Published:** 2021-08-23

**Authors:** Stacey Orangi, Jessie Pinchoff, Daniel Mwanga, Timothy Abuya, Mainga Hamaluba, George Warimwe, Karen Austrian, Edwine Barasa

**Affiliations:** 1 Health Economics Research Unit (HERU), KEMRI-Wellcome Trust Research Program, Nairobi P.O. Box 43640-00100, Kenya; ebarasa@kemri-wellcome.org; 2 Institute of Healthcare Management, Strathmore University, Nairobi P.O. Box 59857-0200, Kenya; 3 Population Council, New York, NY 10017, USA; jpinchoff@popcouncil.org; 4 Population Council, Nairobi P.O. Box 17643-00500, Kenya; dmwanga@popcouncil.org (D.M.); tabuya@popcouncil.org (T.A.); kaustrian@popcouncil.org (K.A.); 5 KEMRI-Wellcome Trust Research Programme, Kilifi P.O. Box 230-80108, Kenya; mhamaluba@kemri-wellcome.org (M.H.); gwarimwe@kemri-wellcome.org (G.W.); 6 Nuffield Department of Medicine, Oxford University, Oxford OX3 7LG, UK

**Keywords:** COVID-19, vaccination, vaccine hesitancy, vaccine confidence, vaccine hesitancy predictors, Kenya

## Abstract

The government of Kenya has launched a phased rollout of COVID-19 vaccination. A major barrier is vaccine hesitancy; the refusal or delay of accepting vaccination. This study evaluated the level and determinants of vaccine hesitancy in Kenya. We conducted a cross-sectional study administered through a phone-based survey in February 2021 in four counties of Kenya. Multilevel logistic regression was used to identify individual perceived risks and influences, context-specific factors and vaccine-specific issues associated with COVID-19 vaccine hesitancy. COVID-19 vaccine hesitancy in Kenya was high: 36.5%. Factors associated with vaccine hesitancy included: Rural regions, perceived difficulty in adhering to government regulations on COVID-19 prevention, no perceived COVID-19 infection risk, concerns regarding vaccine safety and effectiveness, and religious and cultural reasons. There is a need for the prioritization of interventions to address vaccine hesitancy and improve vaccine confidence as part of the vaccine roll-out plan. These messaging and/or interventions should be holistic to include the value of other public health measures, be focused and targeted to specific groups, raise awareness on the risks of COVID-19 and effectively communicate the benefits and risks of vaccines.

## 1. Introduction

The Coronavirus disease (COVID-19) caused by the novel severe acute respiratory syndrome coronavirus-2 (SARS-CoV-2) was declared a pandemic on 11 March 2020 with the first case in Kenya confirmed on the 12th. As of 8 August 2021, there have been 211,828 confirmed COVID-19 cases and 4149 deaths in Kenya [1]. To control the pandemic, non-pharmaceutical interventions (NPIs) have been put in place in Kenya, as in other settings, to slow down the transmission of the virus, thus averting COVID-19 morbidity and mortality. These measures have ranged from physical distancing measures, movement restrictions, closure of schools, sanitation measures, testing and wearing of face masks in public places. These NPIs also have potential indirect effects that are multifaceted, including slow economic growth, financial hardships, reduced access to essential health services, food insecurity, gendered impacts and widening inequality in access to education [2,3]. Given the indirect effects that these NPIs present and based on risk assessments, they have been implemented with different intensities over time in Kenya [3].

Vaccines are a key intervention in the response against the COVID-19 pandemic, with the potential to protect populations from infection, severe disease and death, and block transmission from infected to uninfected people. The development and deployment of COVID-19 vaccines have thus been prioritized [4]. As of 6 August 2021, there were about 294 vaccine candidates in development, the majority of which are in the pre-clinical stages (63%) [5]. There are about 110 vaccines in the clinical phase of development and six vaccines approved for emergency use [5,6].

The government of Kenya plans to vaccinate 50% of all adult populations by the end of June 2022 in a phased approach while maintaining a prioritization matrix. As a result, Kenya launched the rollout of COVID-19 vaccination procured through the COVID-19 Vaccines Global Access facility (COVAX) in March 2021 [7]. The vaccination is being rolled out progressively with the prioritized population being: (1) Essential workers (including healthcare providers, teachers, security personnel), which is an estimated 1.25 million Kenyans. (2) Individuals at risk of severe disease, including older adults (58 years and above) and those above 18 years with co-morbidities—which targets 9.76 million Kenyans. (3) Individuals at high risk of infection, such as people 18 years and above in congregate settings, as well as the hospitality and tourism industry—targeting 4.9 million Kenyans [7]. As of 8 August 2021, there were 1,804,375 COVID-19 vaccine doses administered in Kenya [1].

Given the emergence of new variants that are highly transmissible and reduce vaccine effectiveness, and the inequitable availability of vaccines, there is growing concern that vaccination may not lead to herd immunity [8,9]. However, given that vaccines reduce the risk of severe disease and death, there is consensus that countries will need high levels of vaccine coverage to facilitate a near normal resumption of socio-economic activities, and protect the health system from case surges [9]. One major barrier to achieving high levels of vaccine coverage is vaccine hesitancy, defined as the refusal or delay in acceptance of vaccines, despite their availability [10]. It lies across a spectrum between total acceptance and total refusal [10].

In sub-Saharan Africa, studies suggest that some of the reasons for COVID-19 vaccine hesitancy include negative perceptions of the pharmaceutical industries, concerns on vaccine safety and/or the source of the vaccine, lack of confidence in the government and vaccine costs [11,12,13,14]. Vaccine hesitancy is, however, context-specific and varies across time and place [10]. Kenya reports a high vaccine confidence against childhood diseases, with 89% reporting vaccines as safe, 87% reporting them as being effective and 97% perceived importance for childhood vaccination [15]. However, there is limited evidence and understanding of the public willingness to accept, and the confidence they place on the COVID-19 vaccine in Kenya, which mainly targets the adult population. This study, therefore, aims to determine the level of COVID-19 vaccine hesitancy in Kenya and report on its determinants.

## 2. Materials and Methods

### 2.1. Study Design

This study employed a cross-sectional study design to administer a knowledge, attitudes and practices survey through a phone-based platform. The survey was conducted in February 2021, a month before the deployment of COVID-19 vaccines in Kenya.

### 2.2. Study Population and Setting

The survey was administered to participants sampled from households in four existing Population Council prospective cohort studies across four counties: Kilifi, Kisumu, Nairobi and Wajir [16].

Specifically, in Kilifi County, a coastal region in Kenya, households from three sub-counties (Ganze, Kaloleni and Magarini) enrolled in the Nia Project formed the target population [17]. In Kisumu County, located in Western Kenya, the target populations were households in the Nyalenda area (one of the largest informal settlements in Kisumu) and Kolwa East (a peri-urban area) who were enrolled in the PEPFAR DREAMS study cohort [16]. In Nairobi County the target population was from five urban informal settlements: 2565 households in Huruma and Kibera enrolled in the Adolescent Girls Initiative-Kenya (AGI-K) study [16,18] and 4519 households in Dandora, Kariobangi and Mathare enrolled in the NISITU program study [16,19]. Lastly, in Wajir County, an arid region in north-eastern Kenya, the target population were households from 79 villages in Wajir East, Wajir West and Wajir South sub-counties enrolled in the AGI-K study [16,18]. This is illustrated in Table 1.

### 2.3. Sample Size and Sampling Procedure

Households with available phone numbers were randomly sampled from the four existing cohorts using a ratio of 1:3 for male to female interviews. Due to the nature of the sampling frame described above, the randomly sampled participants were from households with at least one adolescent. Households that solely constituted adult residents or adults and very young children only were not eligible for inclusion in the initial cohorts and therefore not represented in this study.

### 2.4. Data Collection Tool

The data collection tool was a knowledge, attitudes and practices survey that collected information on (1) socio-demographic background information, (2) the knowledge, attitudes and practices reported by households concerning COVID-19, (3) the barriers to adoption of non-pharmaceutical interventions for COVID-19 prevention, (4) the social, economic, education and health effects of COVID-19 prevention measures on adults and (5) the level and determinants of vaccine hesitancy. The latter being the focus of this study.

The data collection tool was designed using validated measures where possible, such as the WHO SAGE vaccine hesitancy tool, and existing COVID-19 vaccine hesitancy tools [20,21], and was also informed by local Kenyan researchers. Questions on the determinants of vaccine hesitancy were categorized into three broad groups adapted from the WHO SAGE vaccine hesitancy tool [20]. The first category reported on individual and group influences. These included questions on the individual’s perceived risk of getting COVID, the ease of following government regulations, societal perception of having COVID-19, the individual adherence to wearing masks and having ever been tested for COVID and the socio-economic impact of COVID-19 on the individual. The second category of questions on determinants of vaccine hesitancy focused on the context, specifically looking into the trusted sources of information for COVID and the perceived level of community support for COVID-19 prevention measures. Lastly, the third category included vaccine-specific questions such as concerns on the COVID-19 vaccine side effects and effectiveness, access to the vaccination site, fear of needles, being too busy to be vaccinated and religious and cultural reasons for refusing vaccination. These variables are reported in Table S1.

The tools were in English and also translated to Swahili, Dholuo and Somali, and were piloted and administered by local interviewers. Data was collected using Open Data Kit.

### 2.5. Ethical Approval

Ethical approval for this study was obtained from both Population Council Institutional Review Board (p936) and AMREF Ethics and Scientific Review Committee (P803/2020). Before data collection, verbal informed consent was obtained from all participants 18 years and over. Participants were told they could terminate the survey at any time or refuse to answer specific questions. Participants were informed beforehand that they would be reimbursed 100 Kenyan shillings (~US $1) for their time (transferred via M-PESA mobile money).

### 2.6. Data Analysis

The socio-demographic characteristics of the sample population were described by computing descriptive statistics. A cross-tabulation analysis was performed to determine the level of vaccine hesitancy among the respondents’ sociodemographic characteristics using chi-squared tests.

Multilevel logistic regression analyses, accounting for the counties as clusters, were performed to compute the adjusted odds ratio (aOR) with a 95% confidence interval. Vaccine hesitancy was the dependent variable and was dichotomized as either accepting (i.e., very likely or somewhat likely to get the vaccine) or hesitant (showing some level of hesitancy, i.e., somewhat unlikely, very unlikely to get the vaccine or do not know). Socio-demographic characteristics, individual risks and perceptions, contextual factors and vaccine-specific issues were included as predictor variables for vaccine hesitancy. The description of the dependent and independent variables is illustrated in Table S1. Predictor variables were included in the multilevel model if found to be significant at a 0.05 significance level in the crude logistic regression and multicollinearity of the variables was assessed using variance inflation factors. The significance level was set at <0.05 and Stata version 15.0 (Stata Corporation, College Station, TX, USA) was used for the data analyses.

## 3. Results

### 3.1. Descriptive Statistics Analyses

The socio-demographic characteristics of the 4136 respondents who participated in this study are shown in Table 2. The mean age of the respondents was 40.8 years (SD 12.6) and the average household size of the respondents was 7.5 (SD 4.4). Most of the respondents were female (67.2%), residents of rural counties (56.0%), married (72.7%), had either no schooling or primary school level education as the highest education level (70.7%) and were from the lowest wealth tertile (36.7%).

**Figure 1:**
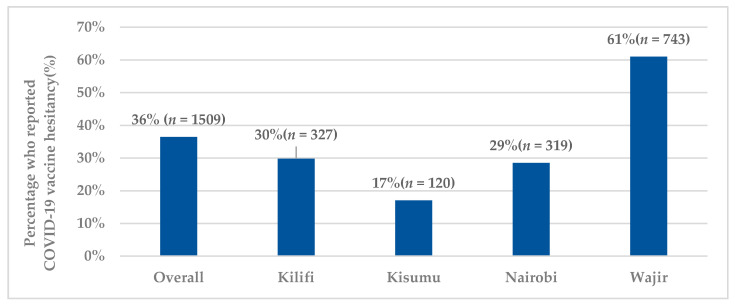
Level of vaccine hesitancy across study counties.

Among the respondents, the amount participants were willing to pay for the vaccine (if it was not available for free) was reported at USD 3.23 (KES 323.06 (SD 1407.69)) and the overall reported hesitancy towards the COVID-19 vaccine was 36.5% (*n* = 1509). The overall level of vaccine hesitancy and across the counties is illustrated in Figure 1.

### 3.2. Bivariate Analysis

Table 3 shows bivariate associations between socio-demographic characteristics and the level of COVID-19 vaccine hesitancy in Kenya. Across all study counties, of the 338 respondents over 58 years, 43.2% (*n* = 146) reported vaccine hesitancy. For those who were married (*n* = 3008), 38.3% (*n* = 1151) were likely to report vaccine hesitancy. Of the 1476 respondents who had no schooling or only pre-primary level of education, 795 (53.9%) reported vaccine hesitancy.

### 3.3. Multilevel Logistic Analysis

The multilevel logistic regression analysis for socio-demographic factors, individual influences, contextual factors and vaccine-specific factors as predictors of vaccine hesitancy among the respondents is illustrated in Table 4. In the multivariate model, respondents who were from rural counties had 2.46 times higher odds of reporting vaccine hesitancy (aOR:2.46; 95% CI:1.02–5.94) as compared to those in urban counties.

Regarding individual influences, those who reported difficulty in adhering to government regulations regarding COVID-19 prevention had 1.96 higher odds of being more likely to be vaccine hesitant (aOR:1.96; 95%CI:1.65–2.33). Similarly, those who reported no perceived COVID-19 infection risk had 1.80 higher odds of being vaccine hesitant (aOR:1.80; 95% CI:1.54–2.10) as compared to those with some perceived COVID-19 infection risk. Individuals who were not socio-economically affected by COVID-19 measures had 1.10 higher odds of being vaccine hesitant (aOR:1.10;95% CI:0.88–1.37) as compared to those who were socio-economically affected, although this was not statistically significant.

In relation to vaccine-specific issues, respondents who were concerned about the COVID-19 vaccine side effects or were concerned about vaccines’ effectiveness had 3.38 higher odds (aOR:3.38; 95% CI:2.81–4.07) and 1.89 higher odds (aOR:1.89; 95%CI:1.17–3.13) of being vaccine hesitant, respectively. Those with religious and cultural reasons had 1.42 higher odds (aOR:1.42; 95% CI:1.01–1.98) of being vaccine hesitant than those with no religious or cultural reasons.

## 4. Discussion

This study set out to evaluate the level and determinants of COVID-19 vaccine hesitancy in Kenya. We make several reflections from the findings. First, the overall level of COVID-19 vaccine hesitancy reported is high (36.5%), as compared to childhood vaccine acceptance in Kenya: Specifically, reported perceived childhood vaccine safety, effectiveness, and relative importance of common childhood vaccines is greater than 87% in Kenya [15]. This highlights the need for the Kenyan government to prioritize interventions to address vaccine hesitancy and improve vaccine confidence as part of its vaccine roll-out plan, and examine the socio-demographic factors, individual effects and perceived risks, context, and vaccine-specific issues that affect vaccine hesitancy. This level of hesitancy is consistent with findings elsewhere. For instance, a survey done across 19 countries reported 71.5% of the respondents were very likely or somewhat likely to accept an available COVID-19 vaccine that is proven safe and effective, with differences in vaccine acceptance ranging from 90% in China to 55% in Russia [22]. In sub-Saharan Africa, surveys have reported 84.6% of Cameroonians, 52% of South Africans, and 50% of Zimbabweans are hesitant or would reject the COVID-19 vaccine [13,23,24].

Second, we explore demographic and socio-economic factors that determine vaccine hesitancy including age, level of education, and socio-economic status. Although not statistically significant, our findings in relation to age are mixed, with those aged 36–57 years reporting less hesitancy but those aged 58+ reporting a higher likelihood of hesitancy as compared to younger people (18–35 years). Some studies [21,22,25,26] have reported a lower likelihood of vaccine hesitancy among the older age groups. However, findings in settings such as Ireland report those aged 35–44 years as being more likely to be vaccine hesitant or resistant than accepting [26]. We report no statistically significant association in the level of education and vaccine hesitancy, which is similarly reported in other low-and middle-income countries [27]. In contrast, other studies report that a higher level of education was associated with less likelihood of COVID-19 vaccine hesitancy [22,25]. We also report on the no statistically significant association between socio-economic status and vaccine hesitancy; this is likely due to the nature of the sample, which was largely from poor households. Other studies reported less vaccine hesitancy among higher income groups [13,26]. These findings illustrate variations among certain sub-groups in the population and hence the need for focussed messaging and campaigns aimed at specific target groups that are more likely to be vaccine hesitant.

Third, attitudes and level of compliance with other NPI COVID-19 measures appear to be associated with hesitancy. For example, those who reported difficulty in adhering to government regulations regarding COVID-19 prevention were more likely to be vaccine hesitant. Other studies have also reported an increased likelihood of vaccine hesitancy amongst those who are less adherent to measures put in place to control COVID-19 [28]. Reduced adherence could also be linked with reduced trust in institutions or government. Findings from a global survey found that those who reported having trust in their governments were less likely to be vaccine hesitant [22]. This finding highlights the need for public health messaging of vaccination to be holistic and include information about the value of other public health measures. As vaccine rollout in Kenya may take some time, NPI preventive measures must continue to avoid a resurgence of infections.

Fourth, individuals’ perceived risk of COVID-19 and its impact on their lives and livelihood also determined their level of hesitancy. For instance, those who had some perceived COVID-19 infection risk were less likely to be hesitant. This finding is similar to findings in Saudi Arabia and France, where individuals with an increased perceived risk of COVID-19 were less likely to be vaccine hesitant [21,25]. This underlines the need to raise awareness about the consequences and risks of COVID-19 and to effectively communicate the value of vaccines.

Fifth, consistent with other studies, those who had concerns over COVID-19 vaccines’ side effects and effectiveness were more likely to be vaccine hesitant [22,25,28,29]. This highlights the prevailing environment where there is heightened concern about the effectiveness and side effects of COVID-19 vaccines. Our findings emphasize the importance of a holistic, dynamic, transparent and consistent public health messaging in improving vaccine hesitancy. Attention should be placed on building trust in the vaccine [30,31]. Additionally, reassurance of the capabilities of the regulatory bodies in ensuring safety and effectiveness should be emphasized [22]. This should be accompanied by open access, real time safety data at a national and regional level and risk-based assessments that inform decision making.

This study focused on one dimension of access, namely acceptability. However, we recognize that other dimensions of access such as the availability of COVID-19 vaccines ensured through procurement and supply-side factors, and the affordability of the vaccines need to be considered over and above vaccine hesitancy as we aim to achieve high levels of vaccine coverage, and control the pandemic. Further, this study has several limitations. First, the sample was drawn from existing Population Council cohorts whose households all have adolescents and were predominantly from urban informal settlements, rural and marginalized areas. Therefore, the sample is not representative of the four counties included in this study and the results are not generalizable to the full population. Second, the study was cross-sectional and reflects the level and determinants of vaccine hesitancy, as of February 2021. This was before the actual COVID-19 vaccine rollout in Kenya that started in March 2021. Conducting a longitudinal study after vaccine deployment would have provided more information on the change in vaccine hesitancy and its drivers, which could also inform the tailoring of messages over time. Third, the study collected data from respondents via phone calls and therefore excluded those without phones. As a result, there is a likelihood of reporting and selection bias. Fourth, there is also a need for qualitative studies to further explore the drivers and deterrents of COVID-19 vaccine uptake and the factors that may improve or compound COVID-19 vaccine acceptance. Despite these reported weaknesses, the study provides important insights on the COVID-19 vaccine hesitancy in certain locations of Kenya and provides implications to policymakers on possible avenues of improving vaccine hesitancy in Kenya.

## 5. Conclusions

Our findings highlight that almost one year into the pandemic and about a month before vaccine rollout had begun in Kenya, vaccine hesitancy was quite high. As the current vaccine rollout plan will take time, there is also a need to promote holistic public health messaging to ensure that NPI interventions such as face masks, hand washing, physical distancing and hand sanitizer use continue. These behaviours are tied to vaccine hesitancy and confidence, clearly linking the two and the need for cohesive messaging campaigns. We find variation by socio-demographics and perceived risk of COVID-19 infection and economic impacts; tailored messages may be required to reach those with different concerns, levels of education, and other factors. Lastly, there is a critical need for accurate and transparent information from trusted sources to combat misinformation, particularly around vaccine side effects and effectiveness.

## Figures and Tables

**Table 1:** Study population and sample size.

County	Location	Underlying Population Council Cohort	Sample Size
Kilifi	3 sub-counties (Ganze, Kaloleni, Magarini)	Nia Project	*n* = 1096 (657 females)
Kisumu	1 informal settlement (Nyalenda) 1 peri-urban area (Kolwa East)	PEPFAR DREAMS study	*n* = 704 (593 females)
Nairobi	5 Informal Settlements (Kibera, Huruma, Dandora, Kariobangi, Mathare)	Adolescent Girls Initiative-Kenya (AGI-K) study NISITU Program	*n* = 1117 (697 females)
Wajir	79 villages in 3 sub-counties (Wajir East, Wajir West, Wajir South)	Adolescent Girls Initiative-Kenya (AGI-K) study	*n* = 1218 (833 females)

**Table 2:** Socio-demographic characteristics by county.

Socio-Demographic Characteristics	Total Sample	Kilifi County	Kisumu County	Nairobi County	Wajir County
	*n* (%)	*n* (%)	*n* (%)	*n* (%)	*n* (%)
All respondents across all counties	4136 (100%)	-	-	-	-
County:					
Urban county (Nairobi/Kisumu)	1822 (44.0%)	-	704 (100%)	1118 (100%)	-
Rural county (Kilifi/Wajir)	2314 (56.0%)	1096 (100%)	-	-	1218 (100%)
Sex:					
Female	2780 (67.2%)	657 (60.0%)	593(84.2%)	697 (62.4%)	833 (68.4%)
Male	1355 (32.8%)	439 (40.0%)	111 (15.8%)	420 (37.6%)	385 (31.6%)
Age group (years):					
18–35	1348 (33.3%)	258 (24.4%)	350 (50.5%)	471 (42.4%)	269 (22.7%)
36–57	2358 (58.3%)	689 (65.1%)	311 (44.9%)	596 (53.7%)	762 (64.4%)
58+	338 (8.4%)	111 (10.5%)	32 (4.6%)	43 (3.9%)	152 (12.9%)
Marital status:					
Single	1, 128(27.3%)	245 (22.4%)	297 (42.2%)	455 (40.7%)	131 (10.8%)
Married	3008 (72.7%)	851 (77.6%)	407 (57.8%)	663 (59.3%)	1087 (89.2%)
Highest level of education:					
No schooling/Pre-primary	1476 (35.7%)	253 (23.1%)	18 (2.6%)	40 (3.6%)	1165 (95.7%)
Primary school	1450 (35.0%)	625 (57.0%)	323 (45.9%)	470 (42.0%)	32 (2.6%)
Secondary school	904 (21.9%)	165 (15.1%)	268 (38.1%)	462 (41.3%)	9 (0.7%)
Tertiary school	306 (7.4%)	53 (4.8%)	95 (13.5%)	146 (13.1%)	12 (1.0%)
Socio-economic status:					
Tertile 1 (Poorest)	1516 (36.7%)	364 (33.2%)	301 (42.9%)	299 (26.8%)	552 (45.3%)
Tertile 2	1120 (27.1%)	382 (34.9%)	179 (25.5%)	536 (48.0%)	23 (1.9%)
Tertile 3 (Wealthiest)	1496 (36.2%)	350 (31.9%)	222 (31.6%)	281 (25.2%)	643 (52.8%)
Average household size: Mean (sd)					
Urban county	5.8 (3.2)	-	6.4 (3.9)	5.4 (2.6)	-
Rural county	8.9 (4.8)	8.8 (4.8)	-	-	9.0 (4.8)

**Table 3:** Bivariate associations between socio-demographic factors and intent of COVID-19 vaccine uptake among respondents in study counties.

Socio-Demographic Factors	Overall		Kilifi County		Kisumu County		Nairobi County		Wajir County	
	Vaccine	*p*-Value	Vaccine	*p*-Value	Vaccine	*p*-Value	Vaccine	*p*-Value	Vaccine	*p*-Value
	Hesitant *n* (%)		Hesitant *n* (%)		Hesitant *n* (%)		Hesitant *n* (%)		Hesitant *n* (%)	
All respondents	1509 (36.5%)	-	327 (29.8%)	-	120 (17.1%)		319 (28.5%)		743 (61.0%)	
County:										
Urban county (Nairobi/Kisumu)	439 (24.1%)	0.000 *								
Rural county (Kilifi/Wajir)	1070 (46.2%)									
Sex:										
Female	1023 (36.8%)	0.559	214 (32.6%)	0.015 *	104 (17.5%)	0.422	203 (29.1%)	0.589	502 (60.3%)	0.438
Male	486 (35.9%)		113 (25.7%)		16 (14.4%)		116 (27.6%)		241 (62.6%)	
Age group (years):										
18–35	453 (33.6%)	0.003 *	87 (33.7%)	0.344	65 (18.6%)	0.083	140 (29.7%)	0.748	161 (59.9%)	0.189
36–57	878 (37.2%)		201 (29.2%)		52 (16.7%)		167 (28.0%)		458 (60.1%)	
58+	146 (43.2%)		31 (27.9%)		1 (3.1%)		11(25.6%)		103 (67.8%)	
Marital status:										
Single	358 (31.7%)	0.000 *	84 (34.3%)	0.084	54 (18.2%)	0.493	114 (31.7%)	0.056	76 (58.0%)	0.458
Married	1151 (38.3%)		243 (28.6%)		66 (16.2%)		175 (26.4%)		667 (61.4%)	
Highest level of education:										
No schooling/Pre-primary	795 (53.9%)	0.000 *	75 (29.6%)	0.51	2 (11.1%)	0.000 *	6 (15.0%)	0.097	712 (61.1%)	0.09
Primary school	363 (25.0%)		178 (28.5%)		37 (11.5%)		125 (26.6%)		23 (71.9%)	
Secondary school	254 (28.1%)		56 (33.9%)		53 (19.8%)		141 (30.5%)		4 (44.4%)	
Tertiary school	97 (31.7%)		18 (34.0%)		28 (29.5%)		47 (32.2%)		4 (33.3%)	
Socio-economic status:										
Tertile 1 (Poorest)	594 (39.2%)	0.000 *	115 (31.6%)	0.611	47 (15.6%)	0.525	97 (32.4%)	0.206	335 (60.7%)	0.693
Tertile 2	297 (26.5%)		108 (28.3%)		30 (16.8%)		143 (26.7%)		16 (69.6%)	
Tertile 3 (Wealthiest)	618 (41.3%)		104 (29.7%)		43 (19.4%)		79 (28.1%)		392 (60.96%)	

* Significant difference at *p* < 0.05.

**Table 4:** Multilevel logistic regression analysis for factors potentially associated with COVID-19 vaccine hesitancy among respondents in Kenya.

Predictor Variables	aOR (95% CI)	*p*-Value
Socio-Demographic Factors		
County:		
Urban county (Nairobi/Kisumu)	Ref	
Rural county (Kilifi/Wajir)	2.46 (1.02–5.94)	0.046 *
Sex		
Female	Ref	
Male	0.91 (0.77–1.08)	0.301
Age group (years):		
18–35	Ref	
36–57	0.96 (0.81–1.14)	0.645
58+	1.03 (0.76–1.39)	0.835
Marital status:		
Single	Ref	
Married	0.92 (0.76–1.10)	0.367
Education:		
No schooling/Pre-primary	Ref	
Primary school	0.92 (0.69–1.24)	0.59
Secondary school	1.21 (0.87–1.69)	0.25
Tertiary school	1.30 (0.87–1.92)	0.2
Socio-economic status:		
Tertile 1 (Poorest)	Ref	
Tertile 2	0.90 (0.74–1.11)	0.325
Tertile 3 (Wealthiest)	0.93 (0.78–1.10)	0.386
Individual influences, risks, and perceptions		
Perceived COVID infection risk:		
Some risk	Ref	
No risk	1.80 (1.54–2.10)	0.000 *
Perceived ability to adhere to government regulations		
regarding COVID-19 prevention:		
Easy to adhere	Ref	
Difficult to adhere	1.96 (1.65–2.33)	0.000 *
Wearing of masks (now compared to when COVID began):		
Wear masks more or the same	Ref	
Wear masks less	1.09 (0.93–1.27)	0.282
Socio-economic impact of COVID measures:		
Socio-economically affected by measures	Ref	
Not socio-economically affected by measures	1.10 (0.88–1.37)	0.407
Context		
Healthcare providers as a trusted source of information:		
No	Ref	
Yes	0.98 (0.84–1.14)	0.768
Vaccine specific issues		
Vaccine side effects concerns:		
No	Ref	
Yes	3.38 (2.81–4.07)	0.000 *
Don’t think the vaccine is effective:		
No	Ref	
Yes	1.89 (1.58–2.27)	0.000 *
Hard to access vaccination sites:		
No	Ref	
Yes	0.72 (0.58–0.90)	0.004 *
Scared of needles:		
No	Ref	
Yes	0.82 (0.64–1.04)	0.105
Religious and cultural reasons:		
No	Ref	
Yes	1.42 (1.01–1.98)	0.040 *
Too busy to get vaccinated:		
No	Ref	
Yes	1.10 (0.81–1.50)	0.527

* Significant difference at *p* < 0.05.

## Data Availability

The data presented in this study are available from the corresponding author on reasonable request.

## Supplementary Materials

The following are available online at https://www.mdpi.com/article/10.3390/vaccines9080936/s1. Table S1: Description of dependent and independent variables for the multilevel logistic regression.
