# Supplementary material for: Assessing the Level and Determinants of COVID-19 Vaccine Confidence in Kenya
Source: Vaccines (Basel). 2021 Aug 23;9(8):936. doi: 10.3390/vaccines9080936 (PMC8402839; doi:10.3390/vaccines9080936)
Supplement: Supplementary file 1 [file vaccines-09-00936-s001.zip › vaccines-1270530-supplementary.pdf]

Table S1. Description of dependent and independent variables for the multilevel logistic regression.

Description	Categorization	Notes
**Dependent variable**		
**Vaccine Hesitancy**	0: Vaccine Hesitant	Vaccine Hesitant (somewhat unlikely, very unlikely to accept the vaccine, or don't know)
	1: Vaccine Accepting	Vaccine Accepting (very likely, or somewhat likely to accept the vaccine
**Independent variables: Socio-demographic factors**		
**County**	0: Urban county (Nairobi/Kisumu)<br>1: Rural county (Kilifi/Wajir)	
**Sex**	0: Female<br>1: Male	
**Age**	1: 18-35 years<br>2: 36-57 years<br>3: 58+ years	
**Education**	1: No schooling/pre-primary<br>2: Primary education<br>3: Secondary<br>4: Tertiary	
**Marital status**	0: Single<br>1: Married	
**Total household size**	Continuous variable	
**Socio-economic status**	1: Tertile 1 (Poorest)<br>2: Tertile 2<br>3: Tertile 3 (Wealthiest)	Principal component analysis was used to calculate socio-economic status based on (electricity, piped water source, reliable water source, livestock, and mobile phone ownership) and categorized into tertile.
**Independent variables: Individual influences, risk and perceptions**		
**Perceived risk of getting COVID**	0: Some risk (low risk, medium risk, high risk)<br>1: No risk	
**Know someone in your family, neighbourhood or workplace who has been infected with COVID**	1: Know someone who tested positive/suspected of having COVID<br>0: Do not know someone who tested positive/suspected of having COVID	

**Societal perception of having COVID**	0. No perceived stigma<br>1. Perceived stigma	This was a composite variable of the following variables 1) People would stop talking to me 2) People would gossip about me 3) People I know would bring me the food I need 4) People I know would bring me the medicines I need 5) People in the community would treat me badly 6) After I recover from coronavirus, people in the community would still avoid me 7) After I recover from coronavirus, I would not be welcome back into my house by family 8) After I recover from coronavirus, I would not be welcome back at my place of work 9) After I recover from coronavirus, I would not be welcome back to my place of worship 10) After I recover from coronavirus, I would not be welcomed back to school
**Ease of following government regulations on COVID**	0: Easy to follow<br>1: Difficult to follow	Easy (Very easy to follow or somewhat easy to follow)<br><br>Difficult (Somewhat difficult to follow and very difficult to follow)
**Compared to the first few month of COVID, adherence to wearing of face masks over mouth and nose**	0: More or about the same<br>1: Less	
**Tested for Covid-19**	0: Tested for COVID<br>1: Never tested for COVID	
**Socio-economically impacted by COVID-19 measures**	0: Socio-economically affected by measures<br>1: Not socio-economically affected by measures	This is a composite variable of the following variables 1) skipped meals in the past 7 days because of not enough money or food 2) Complete loss of job as a result of COVID 3) Partial job loss as a result of COVID 4) Reduced healthcare access 5) economic status-making more or less than when the pandemic just began
	**Independent variables: Context**	
**Perceived measures taken by the community against COVID-19**	0: Community in support of COVID-19 prevention measures<br>1: Community does not support COVID-19 measures	This is a composite variable of the following variables: 1) Community taking steps to protect themselves and others from COVID-19 2) Community angry about social distancing measures due to COVID-19 3) Community work together to prevent and fight COVID-19
**Government and media as trusted source of information**	0: No<br>1: Yes	This is a composite variable of the following variables 1) Government SMS 2) government television advertisements 3) chief/administrators 4) government radio advertisements 5) television programs/shows 6) govt television adverts 7) radio programs/shows 8) internet 9) posters/print adverts 10) social media
**Social Networks as trusted source of information**	0: No<br>1: Yes	This is a composite variable of the following variables: 1) Friends 2) acquaintances/neighbours 3) work colleagues
**Healthcare providers as trusted source of information**	0: No<br>1: Yes	This was a composite variable of the following variables: 1) Public health facility 2) private health facility 3) NGO provider 4) pharmacy 5) community health worker
**Community as trusted source of information**	0: No<br>1: Yes	This was a composite variable of the following variables: 1) public announcements with megaphones 2) church/mosque/sheikh/religious leader 3) community meetings/spaces
	**Independent variables: Vaccination specific issues**	
**Worry about side effects**	0: No<br>1: Yes	This was a composite variable combining: 1) vaccine trust 2) afraid of getting COVID after vaccination 3) worried about side effects
**Don't think the vaccine will be effective**	0: No<br>1: Yes	

**Too busy to get vaccinated**	0: No<br>1: Yes
**Hard to access vaccination site**	0: No<br>1: Yes
**Scared of needles**	0: No<br>1: Yes
**For religious and cultural reasons**	0: No<br>1: Yes
